# Ultrasonographic scoring system for SOS/VOD in pediatric hematopoietic stem cell transplant recipients

**DOI:** 10.1007/s12185-025-03995-1

**Published:** 2025-04-26

**Authors:** Mutsumi Nishida, Shinsuke Hirabayashi, Takahito Iwai, Megumi Sato, Yusuke Kudo, Satomi Omotehara, Tatsunori Horie, Ryosuke Sakano, Yukayo Terashita, Yuko Cho, Atsushi Manabe, Takanori Teshima

**Affiliations:** 1 Management Strategy Department, Hokkaido University Hospital, N14 W5, Kita-ku, Sapporo, 060-8648 Japan; 2 Department of Pediatrics, Hokkaido University Hospital, Sapporo, Japan; 3 Diagnostic Center for Sonography, Hokkaido University Hospital, Sapporo, Japan; 4 Division of Laboratory and Transfusion Medicine, Hokkaido University Hospital, Sapporo, Japan; 5 Department of Radiological Technology, Hokkaido University Hospital, Sapporo, Japan; 6 Department of Pediatrics, Hokkaido University Graduate School of Medicine, Sapporo, Japan; 7 Department of Hematology, Hokkaido University Graduate School of Medicine, Sapporo, Japan

**Keywords:** Children, SOS/VOD, Ultrasonography, Diagnosis, Hematopoietic stem cell transplantation

## Abstract

Sinusoidal obstruction syndrome (SOS)/veno-occlusive disease (VOD) is a serious complication following hematopoietic stem cell transplantation. It is more common in children, with an incidence of approximately 20% compared to 10% in adult patients. While the Hokkaido ultrasonography (US)-based scoring system (HokUS-10 and HokUS-6) has proven effective in diagnosing SOS/VOD in adults, its utility in children remains uncertain. This study evaluated the utility of the HokUS-10/6 scoring systems in diagnosing pediatric SOS/VOD (pSOS/VOD) in a cohort of 99 patients, including 13 diagnosed with pSOS/VOD. Hyperbilirubinemia (> 2 mg/dL) was observed in only three patients (23%), whereas transfusion-refractory thrombocytopenia, hepatomegaly, and ascites were observed in all 13 patients. When the cutoff values for HokUS-10 and HokUS-6 scores were set at 4 and 2, the respective areas under the curve were 0.977 and 0.957. The sensitivity and specificity were 100% and 80% for HokUS-10, and 77% and 97% for HokUS-6, respectively. The simplified HokUS-6 may be a practical tool for diagnosing pSOS/VOD when use of the HokUS-10 scoring system is not feasible. HokUS-10/6 is important for early detection of pSOS/VOD during regular examinations and plays a valuable role in the diagnosis of clinical pSOS/VOD.

**Supplementary Information:**

The online version contains supplementary material available at 10.1007/s12185-025-03995-1.

## Introduction

Sinusoidal obstruction syndrome (SOS), also known as veno-occlusive disease (VOD), is a potentially life-threatening complication that can occur following hematopoietic stem cell transplantation (HSCT) [1]. The complication is more common in children than in adults with an incidence of approximately 20% compared to 10% in adult patients [2], [3]. In accordance with the modified Seattle [4], Baltimore [5], and European Society for Blood and Marrow Transplantation (EBMT) criteria [6], classical SOS/VOD is defined as occurring within 21 days post transplantation. In contrast, late-onset SOS/VOD, as defined by the EBMT [6], occurs at 22 days or after transplantation in adult patients. Pediatric SOS/VOD (pSOS/VOD), also proposed by the EBMT, it is diagnosed when two or more of the clinical manifestations are observed, including transfusion-refractory thrombocytopenia, hepatomegaly, greater than 5% weight gain, ascites, and hyperbilirubinemia (more than 2 mg/dL within 72 h) [3]. Pediatric criteria do not specify a timeframe relative to HSCT. Although hepatomegaly and ascites are recommended to confirm by imaging, other findings of ultrasonography (US) have not yet been established as a diagnostic criterion for pSOS/VOD.

We previously developed ultrasonography-based scoring systems for assessing classical and late-onset SOS/VOD: the Hokkaido US-based scoring system (HokUS-10) [7] and its refined version (HokUS-6) (Table 1) [8]. In this study, our objective was to evaluate the diagnostic performance of the HokUS-10/6 scoring systems for pSOS/VOD.

**Table 1:** Ultrasonographic scoring system of HokUS-10 and 6

US scoring system		HokUS-10	HokUS-6*
Positive cut off value of SOS/VOD		≧ 5	≧ 2
Parameters	Description	Points	
Hepatic left lobe vertical diameter^†^	≧ 70 mm	1	–
Hepatic right lobe vertical diameter^†^	≧ 110 mm	1	–
Gall bladder wall thickening	≧ 6 mm	1	1
PV diameter^†^	≧ 12 mm	1	1
PUV diameter	≧ 2 mm	2	–
Amount of ascites	A little	1	–
	Moderate to severe	2	1
PV mean velocity	< 10 cm/s	1	1
Direction of portal vein flow	Congestion or hepatofugal	1	–
Appearance of PUV blood flow signal	Yes	2	1
Hepatic artery RI^‡^	≧ 0.75	1	1
Max scores		13	6

*PV* portal vein, *PUV* para-umbilical vein, RI resistive index

*Presence of ascites was mandatory, ^†^The cut off values of hepatomegaly [10] and PV diameter [11] in pediatric patients, ^‡^RI was calculated by Vmax-Vmin/Vmax

## Patients and methods

One hundred eight patients who underwent HSCT from June 2008 to January 2023 at Hokkaido University Hospital were retrospectively analyzed.

pSOS/VOD was clinically diagnosed, and its severity was defined according to the pediatric EBMT (pEBMT) criteria [3]). Patients who did not meet the pEBMT criteria served as the control group. The severity was divided into five categories: grade 1 = mild; grade 2 = moderate; grade 3 = severe; grade 4 = very severe; and grade 5 = death. Acute graft-versus-host disease (aGVHD) was diagnosed according to the guidelines of the consensus conference on aGVHD grading [9].

This study was approved by the institutional review board (IRB#023–0111), and research consent was obtained via an opt-out approach.

### US evaluation

US was performed before conditioning therapy (pre-HSCT), mainly 14 and 28 days after HSCT, and at the time when pSOS/VOD was clinically suspected, as reported in previous studies [7], [8].

US evaluations were performed via convex transducers (center frequency, 3.75–6 MHz) and linear transducers (center frequency, 7.5 MHz) equipped with Aplio™ XG/500/i700/i800 and Viamo/Xario (Canon Medical Systems Corp., Otawara, Japan) by 7 medical sonographers with more than 5 years of experience. The HokUS-10 scoring system consists of 10 parameters: (1) hepatomegaly in the left lobe, (2) hepatomegaly in the right lobe, (3) dilatation of the main portal vein (PV), (4) hepatofugal flow in the main PV, (5) decreased velocity of the PV, (6) dilatation of the paraumbilical vein (PUV), (7) a blood flow signal in the PUV, (8) gallbladder (GB) wall thickening, (9) ascites, and (10) an increased resistive index of the hepatic artery (HA-RI) (Table 1) [7]. Ascites was graded into 3 levels: none, a little, and moderate to severe. A moderate amount of ascites was originally defined by the presence of ascites in all spaces in the subhepatic, spleno-renal interspace, and Douglas pouch (maximum thickness ≥ 1 cm for at least at the 2 sites). The methods used to diagnose hepatomegaly [10] and measure the PV diameter[11] in pediatric patients are shown in Supplementary Figure, and cutoff values were evaluated according to previous reports (Table 2) [10], [11].

**Table 2:** Liver size and portal vein diameter in children

(A) The left and right liver lobe sizes [10]						
Age	Left lobe (mm) mean	SD (mm)	Upper limit (mm)	Right lobe (mm) mean	SD (mm)	Upper limit (mm)
0–1-month-old	71.5	8.6	80.1	55.7	6.9	62.6
2–5-month-old	75.4	9.4	84.8	60.9	5.3	66.2
6–11 month-old	82.8	8.2	91.0	65.0	8.5	73.5
1 year-old	91.9	11.1	103.0	75.5	7.8	83.3
2-year-old	100.1	11.1	111.2	81.4	6.8	88.2
3-year-old	108.0	9.7	117.7	88.1	7.4	95.5
4-year-old	110.2	9.2	119.4	84.7	4.9	89.6
5-year-old	114.9	9.5	124.4	91.5	7.4	98.9
6-year-old	121.8	8.3	130.1	93.5	6.6	100.1
7-year-old	125.0	7.3	132.3	94.1	7.4	101.5
8-year-old	121.8	8.3	130.1	99.4	6.1	105.5
9-year-old boy	133.6	7.8	141.4	99.1	9.5	108.6
9-year-old girl	121.2	9.0	130.2	99.7	8.8	108.5
10-year-old boy	128.4	10.7	139.1	103.0	6.8	109.8
10-year-old girl	129.1	10.0	139.1	107.8	10.4	118.2
11-year-old boy	132.5	11.7	144.2	108.9	7.3	116.2
11-year-old girl	131.8	16.0	147.8	103.2	12.4	115.6
12-year-old boy	138.6	9.4	148.0	111.0	10.9	121.9
12-year-old girl	134.9	9.4	144.3	113.2	8.7	121.9
13-year-old boy	144.0	7.0	151.0	115.9	9.3	125.2
13-year-old girl	135.6	7.8	143.4	111.8	10.7	122.5
14–15-year-old boy	144.2	17.0	161.2	125.6	6.9	132.5
14–15-year-old girl	135.2	12.2	147.4	110.0	9.1	119.1

(B) The portal vein diameter [11]		
Age	Diameter (mm)	Cut off value (mm)
0–5-year-old	4–8	≧ 8
6–10-year-old	6–9	≧ 9
11–15-year-old	7–11	≧ 11

Upper limit (+ 1SD or higher) is considered hepatomegaly and scored accordingly

*SD* standard deviation

Table 2A and B have been adapted with modifications

The HokUS-6 scoring system evaluates 6 parameters selected from the 10 parameters of HokUS-10 (Table 1) [8]. The HokUS-6 system is used only when ascites is present, and 6 parameters are evaluated: the amount of ascites, the appearance of the PUV blood flow signal, GB wall thickening, PV dilatation, the decrease in the PV velocity, and the increase in HA-RI.

### Statistical analysis

A receiver operating characteristic (ROC) curve was used to evaluate the diagnostic accuracy. The differences between the HokUS-10 and HokUS-6 scoring systems were evaluated via the McNemar test. A desirable diagnostic performance can be indicated with confidence intervals (CIs). The correlations of the HokUS-10 and HokUS-6 scores with the severity of pSOS/VOD were evaluated via Spearman’s rank correlation coefficient (correlations are indicated as “ρ”). The Tukey‒Kramer honestly significant difference test was used to compare HokUS-10 and HokUS-6 scores between groups. The prediction of pSOS/VOD was evaluated via the Cochran‒Armitage trend test. Statistical analysis was performed via standard statistical software (JMP Pro Version 17.2.0; SAS Institute Inc., Cary, NC, USA, and R [version 4.3.0. http://www.Rproject.org/]).

## Results

### Patient characteristics

Following the exclusion of 9 patients due to insufficient data, a total of 99 patients who underwent at least one US examination within 120 days post-HSCT were included in this study. The median age of these 99 patients was 5 years (range: 0–16 years). The characteristics of the patient cohort are summarized in Table 3. The cohort comprised 18 patients with acute lymphoblastic leukemia, 14 patients with acute myeloid leukemia, 27 patients with solid tumors, and 17 patients with inborn errors of immunity. Among these patients, 20 patients (20%) had a previous history of allogeneic HSCT. Myeloablative conditioning (MAC) and reduced-intensity conditioning (RIC) regimens were administered to 68 and 31 patients, respectively. All patients received prophylaxis for pSOS/VOD with ursodeoxycholic acid and low-molecular-weight heparin. Notably, two patients did not undergo US examination prior to HSCT. No significant differences were observed in patient characteristics between the pSOS/VOD group and the control group.

**Table 3:** Patient characteristics

Characteristics	Total	SOS/VOD	Control	P value
Number of patients	99	13	86	
Median age (range)	5 (0–16)	7 (0–14)	5 (0–16)	0.880
Male/female	65/34	10/3	55/31	0.359
Disease				
ALL	18	3	15	0.370
AML	14	0	14	
Other leukemia/lymphoma^†^	6	1	5	
Solid tumors^‡^	27	3	24	
Brain tumors^§^	7	1	6	
Inborn errors of immunity^||^	17	5	12	
Metabolic disorders^¶^	5	0	5	
Bone marrow failure^⁑^	5	0	5	
Prior history of HSCT	20	2	18	0.647
Stem cell source				
BM/PB/CB	23/36/40	2/7/4	21/29/36	0.475
Donor				
Related/unrelated/auto	14/56/29	4/5/4	10/51/25	0.144
Disease status at HSCT				
CR/non-CR	43/29	4/4	39/25	0.865
Conditioning regimen				
MAC/RIC	68/31	8/5	60/26	0.436
TBI-containing (10Gy≦)/busulfan	46	4	42	0.224
Use of busulfan	36	7	29	0.160

*ALL* acute lymphoblastic leukemia, *AML* acute myeloid leukemia, *HSCT* hematopoietic stem cell transplantation, *CR* complete remission, *BM* bone marrow, *PB* peripheral blood stem cell, *CB* cord blood, *PB* peripheral blood stem cell, *CB* cord blood, *RIC* reduced-intensity conditioning, *MAC* myeloablative conditioning, *TBI* total body irradiation

^†^Other leukemia/lymphoma: Blastic Plasmacytoid Dendritic Cell Neoplasm [2], Juvenile Myelomonocytic Leukemia [1], Lymphoma [2], Acute undifferentiated leukemia [1] ^‡^Solid tumors: Neuroblastoma [18], Rhabdomyosarcoma [3], Malignant rhabdoid tumor [3], Ewing sarcoma [1], Pleuropulmonary blastoma [1] Choriocarcinoma [1], ^§^Brain tumors: Atypical teratoid/rhabdoid tumor [3], Medulloblastoma [3], Retinoblastoma [1], ^||^Inborn errors of immunity: Wiskott-Aldrich syndrome [3], Chronic granulomatous disease [6], CD40 ligand deficiency [3], X-linked severe combined immunodeficiency [1], Nuclear factor-κB essential modulator abnormality [1], Signaling lymphocyte activation molecule-associated protein deficiency [2], Familial hemophagocytic lymph histiocytosis; [1], ^¶^Metabolic disorders: Adrenoleukodystrophy [5], ^⁑^Bone marrow failure: pure red cell aplasia [1], Fanconi anemia [1], Anaplastic anemia [3]

### Clinical diagnosis

Thirteen patients (13%) were diagnosed with pSOS/VOD, with the median day of clinical diagnosis being 28 days post-HSCT (range: 3–105 days; Table 4). Of whom, two patients (15%) met the classical criteria for pSOS/VOD, whereas the remaining 11 patients (85%) met two or more of the pEBMT criteria in conjunction with ultrasound findings. Hyperbilirubinemia (> 2 mg/dL) was observed in only three patients (23%), whereas all 13 patients exhibited transfusion-refractory thrombocytopenia, hepatomegaly, and ascites. Early diagnosis was made in case #7 and case #13, in case #7 the HokUS-10 and HokUS-6 detected pSOS/VOD 5 days before the clinical diagnosis. However, in case #13, only HokUS-10 enabled early diagnosis, identifying the condition 7 days prior to clinical diagnosis. In the remaining patients, the clinical diagnosis was confirmed on the same day as ultrasound detection. Five of the pSOS/VOD patients (39%) died within 360 days following HSCT, of whom one patient (8%) died due to pSOS/VOD. Details of the pSOS/VOD patients are shown in Table 5.

**Table 4:** Details of the patients who developed pediatric SOS/VOD

Number of patients	13
A median (range) day after HSCT	
To clinical diagnosis To US scoring system (HokUS-10) (HokUS-6)	28 (3–105) 28 (0–105) 28 (0–105)
EBMT severity grading; 1/2/3/4/5	3/0/2/7/1
Comorbid aGVHD	1
Death Day of death after HSCT	5 60–145

*HSCT* hematopoietic stem cell transplantation, *EBMT* the European society for blood and marrow transplantation, *aGVHD* acute graft versus host disease

**Table 5:** Details of the patients who developed SOS/VOD

Case No	Age/sex	Disease	Disease status at HSCT	Donor/stem cell source	Conditioning	Engraftment (day)	SOS/VOD clinical diagnosis (day)	HokUS-10 diagnosis (day)	HokUS-10 score at diagnosis	HokUS-6 score at diagnosis	Max HokUS-10 score	Max HokUS-6 score	EBMT severity grade	Presence of MOF	DF	1-year survival (day)
1	12/M	ALL	CR	Mismatch/BM	Flu/Mel/ATG	15	13	14	7	2	7	2	4	Yes	No	Alive
2	6/M	WAS	NA	Mismatch/BM	CY/Bu/ALG	22	21	20	8	3	8	3	4	No	No	Alive
3	14/M	Ewing	Non-CR	Auto/PB	Mel/Bu	11	41	41	7	3	7	3	4	No	No	Death (137) DOD
4	13/F	ALL	Non-CR	Mismatch/PB	Flu/ATG/Mel/TBI	16	73	73	10	4	11	4	4	Yes	No	Death (88) Infection
5	2/M	NB	CR	Auto/PB	Mel/Bu	12	28	28	7	3	7	3	3	Yes	No	Alive
6	10/M	MB	CR	Auto/PB	Mel/VP	10	3	3	5	3	11	5	5	No	No	Death (60) SOS/VOD
7	0/F	AUL	CR	Mismatch/CB	Ful/Mel/CA/TBI	19	5	0	5	2	7	2	4	Yes	Yes	Alive
8	0/M	NEMO	NA	Mismatch/CB	Flu/Mel	11	105	105	7	4	7	4	4	Yes	Yes	Death (145) Infection
9	7/M	ALL	CR	Mismatch/CB	VP/CY/TBI	14	12	12	4	1	4	1	1	No	Yes	Alive
10	11/M	SAP	NA	Mismatch/PB	Aletuzumab/Bu/Flu	21	43	42	4	1	4	1	1	No	Yes	Alive
11	2/F	NB	Non-CR	Auto/PB	Bu/Mel	11	44	44	8	4	8	4	3	No	Yes	Death (80) DOD
12	9/M	XCGD	NA	Mismatch/PB	Flu/Bu/ATG/TBI	14	35	35	9	3	9	3	1	No	Yes	Alive
13	0/M	FHL	NA	Mismatch/CB	Flu/Bu	14	14	7	4	1	9	5	4	Yes	Yes	Alive

*ALL* acute lymphoblastic leukemia, *WAS* Wiskott-Aldrich syndrome, *Ewing* Ewing sarcoma, *NB* neuroblastoma, *MB* cerebellar medulloblastoma, *AUL* acute undifferentiated leukemia, NEMO nuclear factor -κB essential modulator abnormality, *SAP* Signaling lymphocyte activation molecule-associated protein deficiency, *XCGD* Chronic granulomatous disease, *FHL* familial hemophagocytic lymphohistiocytosis, *CR* complete remission, *BM* bone marrow, *PB* peripheral blood stem cell, *CB* cord blood, *Flu* fludarabine, *Mel* Melphalan, *ATG* antithymocyte globulin, *CY* cyclophosphamide, *Bu* Busulfan, *ALG* antilymphocyte globulin, *TBI* total body irradiation, *VP* Etoposide, *CA* cytarabine, *MOF* multiple-organ failure, *DF* Defibrotide, *DOD* death of primary disease

### US findings

A total of 377 US examinations were performed from the pre-HSCT period to 120 days post-HSCT. On average, each patient underwent 3.8 US examinations (range: 2–11). The US diagnosis was made either before or on the same day as the clinical diagnosis in all patients, with a median time of 28 days post transplantation (range: 0–105 days), via the HokUS-10 and HokUS-6 systems (Table 4). Positive cutoff values were observed in 30 patients for whom the HokUS-10 system was used and in 13 patients for whom the HokUS-6 system was used. Among these patients, 13 patients were diagnosed with pSOS/VOD by pEBMT along with the HokUS-10 system, and 10 patients were diagnosed by pEBMT along with the HokUS-6 system (Table 6). The numbers of true-positive, true-negative, false-negative, and false-positive results were 13, 69, 0, and 17 for the HokUS-10 system and 10, 83, 3, and 3 for the HokUS-6 system, respectively.

**Table 6:** Comparison of clinical diagnosis and US scoring systems of SOS/VOD in pediatric patients

Clinical diagnosis	US scoring system			
	HokUS-10		HokUS-6	
	≧ 4	< 4	≧ 2	< 2
Yes	13	0	10	3
No	17^†^	69	3	83

^†^Three patients diagnosed via the HokUS-10 system were treated with recombinant human soluble thrombomodulin after the score increased above the cutoff value

**Fig. 1:**
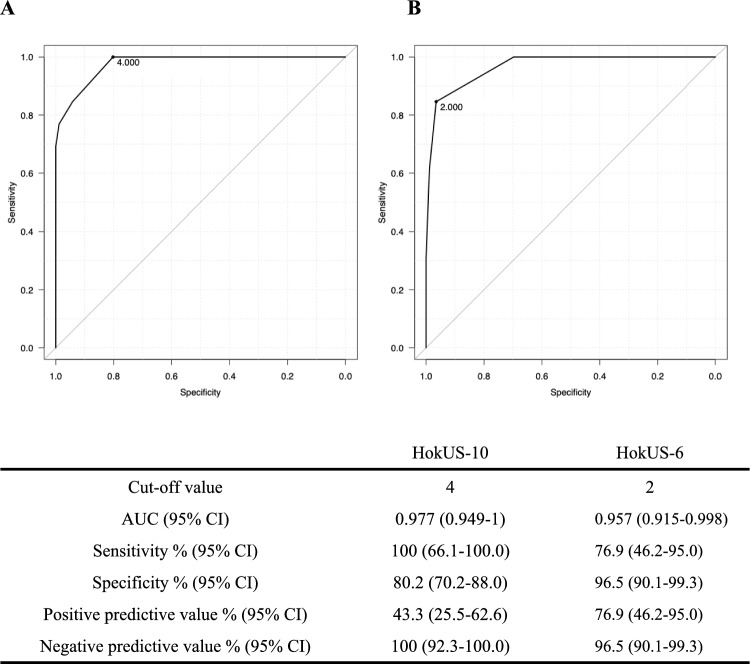
Areas under the ROC curve calculated with the HokUS-10 and HokUS-6 systems and their diagnostic performance. The upper part shows the areas under the curve (AUCs) of the HokUS-10 and HokUS-6 systems. (A) HokUS-10 and (B) HokUS-6 scoring system. The diagnostic values of HokUS-10 and HokUS-6 are shown below (N = 99). AUC; area under the curve, CI; confidence interval

**Fig. 2:**
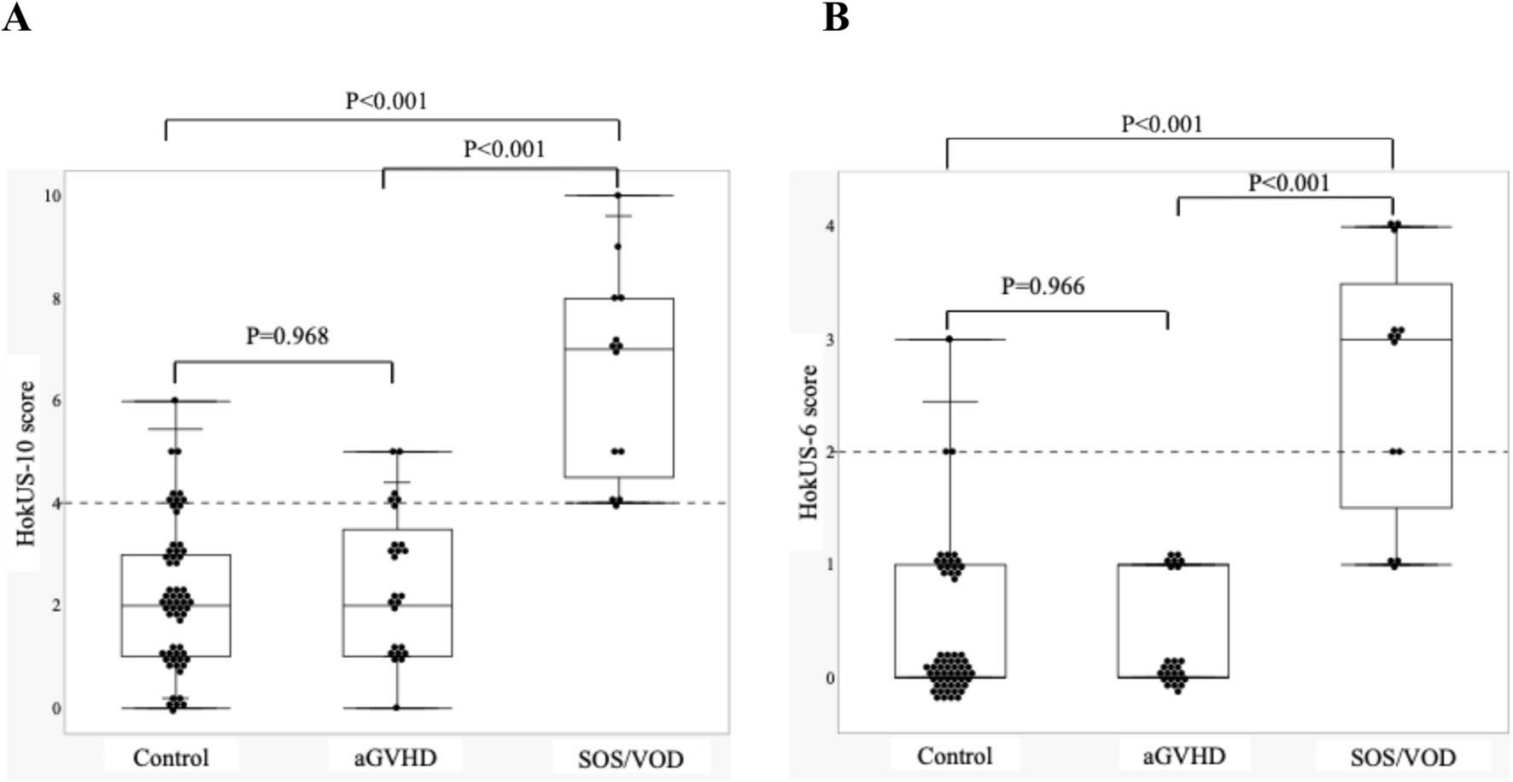
HokUS-10 and HokUS-6 scores in the control, aGVHD and SOS/VOD groups following HSCT. Controls were classified as having neither SOS/VOD nor aGVHD, and the scores represent the highest values observed. The lines in each graph are the maximum, upper quartile, median, lower quartile, and minimum from the top. (A) HokUS-10 and (B) HokUS-6 scoring system. aGVHD: acute graft-versus-host disease

The area under the curve (AUC) was 0.977 (95% CI: 0.949–1) for the HokUS-10 system and 0.957 (95% CI: 0.915–0.998) for the HokUS-6 system (Fig. 1), with no significant difference between the two (p = 0.165). The sensitivity, specificity, positive predictive value, and negative predictive value for diagnosing pSOS/VOD were 100%, 80%, 43%, and 100%, respectively, for the HokUS-10 system and 77%, 97%, 77%, and 97%, respectively, for the HokUS-6 system (Fig. 1). False-positives were observed in 17 patients diagnosed via the HokUS-10 system and in 3 patients diagnosed via the HokUS-6 system. Among the false-positives obtained with the HokUS-10 system, the clinical diagnoses included acute graft-versus-host disease (aGVHD) (6 cases), infections (3 cases), engraftment syndrome (1 case), and others (7 cases). False positives with HokUS-6, the clinical diagnosis were others in all three cases. Notably, HokUS-10 and HokUS-6 scores were significantly lower in 25 aGVHD (not of the liver but in general) patients without pSOS/VOD who underwent US within 7 days after onset of aGVHD than in patients with pSOS/VOD (both p < 0.001, Fig. 2).

The distribution of pSOS/VOD severity among the patients was as follows: grade 1 (3 patients), grade 2 (0 patients), grade 3 (2 patients), grade 4 (7 patients), and grade 5 (1 patient). Ten (77%) patients were classified as Grade 3 or higher. The maximum HokUS-10 scores were not significantly correlate with the EBMT severity grading of pSOS/VOD (ρ = 0.478, p = 0.098), and whereas, the maximum HokUS-6 scores showed a significantly correlation (ρ = 0.563, p = 0.045); see Supplementary Table.

**Fig. 3:**
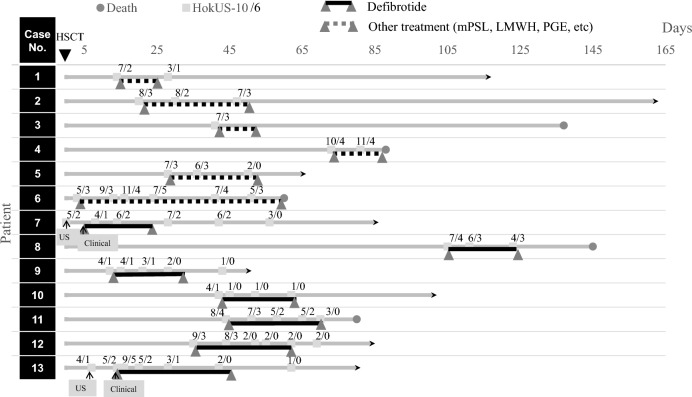
Treatment period and follow-up study of the HokUS-10/6 scoring system for pSOS/VOD. Early detection was successfully accomplished via the HokUS-10 scoring system for patients #7 and #13. Especially, in case 13, only HokUS-10 enabled early diagnosis. Among the patients who survived, HokUS-10 and HokUS-6 scores steadily decreased to two or one and 0, respectively, following the initiation of treatment. Conversely, patients who did not survive presented an inadequate reduction in HokUS-10 and HokUS-6 scores, which tended to remain three or higher. mPSL; methylprednisolone, LMWH; low molecular weight heparin, PGE; prostaglandin

Figure 3 illustrates the treatment period and follow-up study for pSOS/VOD as assessed by the HokUS-10/6 system.

In the pre-HSCT setting, significant differences were observed between the pSOS/VOD and control groups based on HokUS-6 scores (p = 0.038); however, no significant differences were found with HokUS-10 scores (p = 0.108).

## Discussion

The HokUS-10 scoring systems showed excellent diagnostic performance in diagnosing pSOS/VOD using the decreased cutoff value of 4. The HokUS-6 scoring system showed rather low sensitivity but high specificity using same cutoff value of 2 which we demonstrated in our previous studies of SOS/VOD [8], [12].

Hyperbilirubinemia is not required for the diagnosis of pSOS/VOD; thus, 10 (77%) patients in this study did not have hyperbilirubinemia. In pEBMT, the diagnosis of pSOS/VOD is determined by the presence of two or more of the following: transfusion-refractory thrombocytopenia, hepatomegaly, a weight gain exceeding 5%, ascites, and/or hyperbilirubinemia. The primary aim of pEBMT was to facilitate early diagnosis and intervention with defibrotide. This approach was also applied to the pediatric HokUS-10/6 (pHokUS-10/6) systems in this study. Consequently, the pHokUS-10 cutoff value was decreased from 5 (used in adults) to 4. In two patients, the US diagnosis was made prior to the clinical diagnosis via decreased cutoff value, whereas in the other patients, diagnoses were made on the same day. When we used same cutoff value of 5 in HokUS-10, sensitivity declined to 77%, because of three patients showed false negativity. Although this lower cutoff value of 4 resulted in an increased number of false positives, it enabled the detection of early hemodynamic changes in the liver indicative of sinusoidal obstruction via US.

Three patients were false-negative by HokUS-6 compared to HokUS-10. In cases #9 and #10, HokUS-10 yielded true-positive results, whereas HokUS-6 showed false-negative findings. Their HokUS-6 score was 1 (below the cutoff value), suggesting mild pSOS/VOD, which rapidly improved following the initiation of defibrotide treatment on the day of HokUS-10 diagnosis, as shown in Fig. 3. Importantly, no significant adverse outcomes were observed in these patients. In case #13, a clinical diagnosis was not established on the day of the HokUS-10 diagnosis. However, the patient met the pEBMT criteria after seven days. This patient had familial hemophagocytic lymphohistiocytosis (FHL), which is characterized by hepatosplenomegaly, potentially affecting the US findings. HokUS-10/6 were instrumental in confirming SOS/VOD and facilitating early diagnosis. When patients present thrombocytopenia, evaluation using HokUS-10/6 is recommended to differentiate other entities of thrombocytopenia.

US is often considered operator dependent. To address this challenge, we assessed inter-operator reliability in the evaluation of HokUS-10 among three sonographers with varying levels of experience in adult patients [13]. The agreement rate and Krippendorff’s alpha coefficient for HokUS-10 among operators with 2, 7, and 33 years of experience were 100% and 0.63, respectively. These findings suggest that HokUS-10 is a reliable tool for diagnosing SOS/VOD, even when used by less experienced operators.

Also, in US diagnosis, evaluating PV mean velocity and HA-RI can sometimes be challenging. To address this issue, we developed a modified HokUS-10/6, which incorporates a right intercostal scan to minimize interference from intestinal gas and improve image quality [14]. The modified HokUS-10/6 facilitates assessment not only in adults but also in children. Additionally, adjusted cutoff values were determined in the prospective study.

In this study, pSOS/VOD occurred at median of 28 days post-transplant, in line with other reports. A review article of SOS/VOD in both pediatric and adult patients showed that characteristic clinical features were detected in first no consistent pattern approximately 30 to 50% of patients, and that differentiating SOS/VOD from aGVHD remains a significant challenge [15]. However, in the HokUS-10/6 scoring systems, the scores for pSOS/VOD are significantly higher than those for aGVHD. In consequence, HokUS-10/6 scoring systems will help diagnosis pSOS/VOD in patients whose diagnosis is difficult. Currently, some criteria for diagnosis of pSOS/VOD are available, such as the pEBMT criteria [3] and Cairo criteria [16]. Incorporating HokUS-10/6 into these diagnostic frameworks will offer a more comprehensive approach to identifying pSOS/VOD, thereby enhancing patient outcomes through timely and precise diagnosis.

Recently, the usefulness of a variety of elastography has been reported as one of diagnostic tools of SOS/VOD [17]. Elastography is noninvasive and easy, however evaluated area is limited, usually at one cross section and sampling area of point shear wave is only a region 5 × 15 mm in the liver [18]. While HokUS-10/6 could estimate blood flow alteration of the entire liver. Indeed, diagnostic performance of elastography was not high enough compared to HokUS-10/6, AUC was 0.779 [19] versus 0.977 by HokUS-10.

As our previous study showed correlation of severity of classical SOS/VOD [8], HokUS-6 showed correlation of EBMT severity grading. However, HokUS-10 scores were not correlated with the EBMT severity grading in this cohort of children. HokUS-6 may contribute to estimate the clinical severity of pSOS/VOD.

In follow-up studies, persistently high HokUS-10/6 scores after treatment initiation were associated with increased mortality, whereas a marked reduction tended to correlate with improved survival. Notably, a HokUS-6 score of 0 may indicate a favorable treatment response (Fig. 3). Therefore, the HokUS-10/6 system may serve as a valuable tool not only for diagnostic purposes but also for evaluating disease progression.

Our previous study showed pre-HSCT HokUS-6 scoring in SOS/VOD patients were significantly higher than those in non-pSOS/VOD patients. Although pre-HSCT HokUS-6 scores in this study might offer some potential in predicting the onset of pSOS/VOD, the statistical significance observed is only marginal, which limits the strength of the evidence. To fully establish the severity grading and predictive value of both the HokUS-10 and HokUS-6 scoring systems, further research with larger sample sizes is required. In fact, a prospective, larger-scale, multicenter study is currently underway to validate our findings in Japan.

In conclusion, the simplified HokUS-6 may offer a practical tool for diagnosing pSOS/VOD when the HokUS-10 scoring system is not available. HokUS-10/6 is important in detecting pSOS/VOD at an early stage during regular examinations as a surveillance method. Additionally, this method is valuable in assisting the diagnosis of clinical pSOS/VOD.

## Supplementary Information

Below is the link to the electronic supplementary material.

Supplementary file1 (DOCX 1336 KB)

Supplementary file2 (DOCX 18 KB)

## Data Availability

The datasets are available from the corresponding author on reasonable request, due to restrictions related to patient privacy and institutional regulations.
