# Supplementary material for: Ultrasonographic scoring system for SOS/VOD in pediatric hematopoietic stem cell transplant recipients
Source: Int J Hematol. 2025 Apr 26;122(3):434–43. doi: 10.1007/s12185-025-03995-1 (PMC12380934; doi:10.1007/s12185-025-03995-1)

**Supplementary Figure. Hepatic lobs and portal vein measurements.**

Left lobe size is the sum of the craniocaudal length (L1) and anteroposterior width (L2) in the longitudinal plane through the aorta (A). Right lobe size is the sum of the distance from the anterior liver surface to the midpoint of the horizontal portion of the portal vein (R1), and from that midpoint to the deepest phrenic surface (R2) using the right subcostal approach (B). Portal vein diameter is measured as the maximal diameter of the main portal vein via the subcostal approach (C).

**A B**


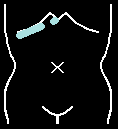

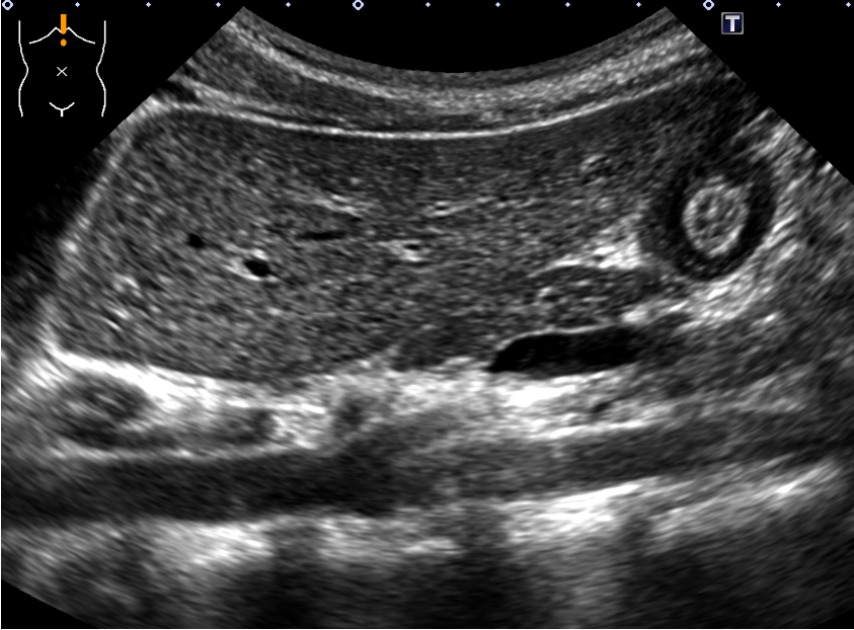

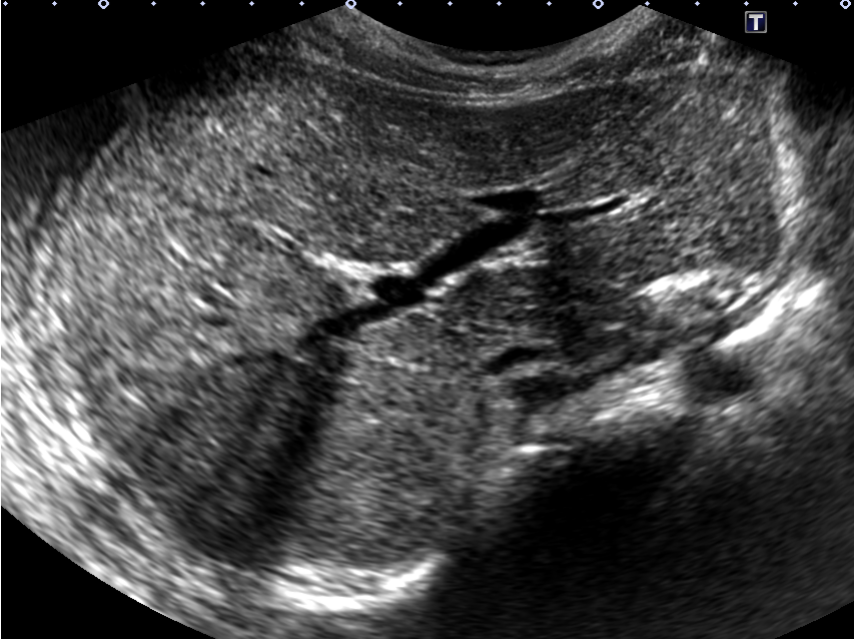


R2

R1

L2

L1

**C**


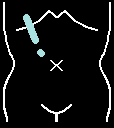

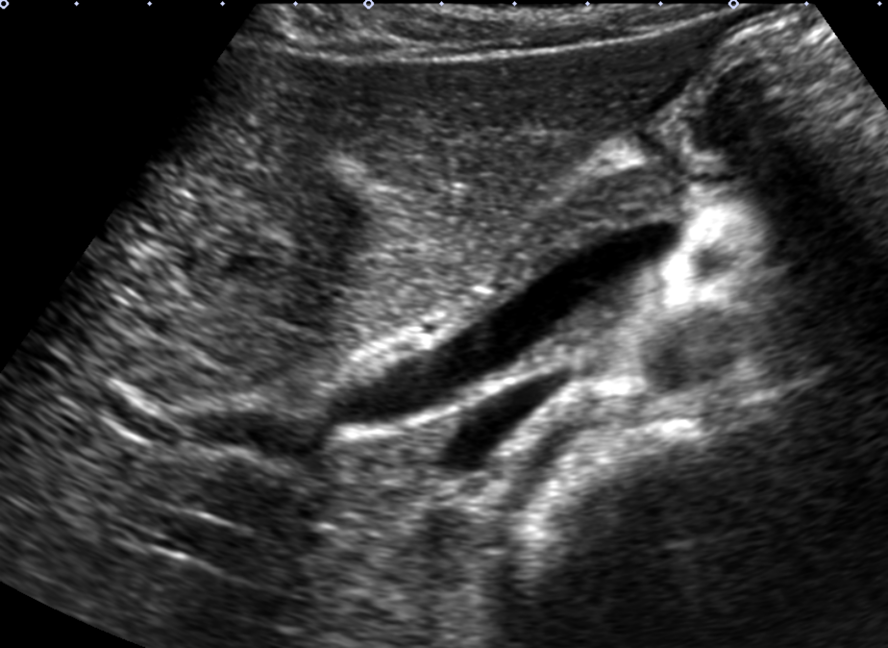

Supplement: Supplementary file 1 — Supplementary file1 (DOCX 1336 KB) [file 12185_2025_3995_MOESM1_ESM.docx]
