# Supplementary material for: Ultrasonographic scoring system for SOS/VOD in pediatric hematopoietic stem cell transplant recipients
Source: Int J Hematol. 2025 Apr 26;122(3):434–43. doi: 10.1007/s12185-025-03995-1 (PMC12380934; doi:10.1007/s12185-025-03995-1)
Supplement: Supplementary file 2 — Supplementary file2 (DOCX 18 KB) [file 12185_2025_3995_MOESM2_ESM.docx]

**Supplementary table.** The correlation of maximum HokUS-10/6 scores and the EBMT severity grading of SOS/VOD

|  | | Max HokUS-10 | | | | | | | |
| --- | --- | --- | --- | --- | --- | --- | --- | --- | --- |
|  |  | 4 | 5 | 6 | 7 | 8 | 9 | 10 | 11 |
| EBMT severity grading | Grade 1 | 2 | 0 | 0 | 0 | 0 | 1 | 0 | 0 |
|  | Grade 2 | 0 | 0 | 0 | 0 | 0 | 0 | 0 | 0 |
|  | Grade 3 | 0 | 0 | 0 | 1 | 1 | 0 | 0 | 0 |
|  | Grade 4 | 0 | 0 | 0 | 4 | 1 | 1 | 0 | 1 |
|  | Grade 5 | 0 | 0 | 0 | 0 | 0 | 0 | 0 | 1 |

|  | Max HokUS-6 | | | | | |
| --- | --- | --- | --- | --- | --- | --- |
|  |  | 1 | 2 | 3 | 4 | 5 |
| EBMT severity grading | Grade 1 | 2 | 0 | 1 | 0 | 0 |
|  | Grade 2 | 0 | 0 | 0 | 0 | 0 |
|  | Grade 3 | 0 | 0 | 1 | 1 | 0 |
|  | Grade 4 | 0 | 2 | 2 | 2 | 1 |
|  | Grade 5 | 0 | 0 | 0 | 0 | 1 |
